# Educating Health Professionals about Drug and Device Promotion: A Nepalese Perspective

**DOI:** 10.1371/journal.pmed.0040089

**Published:** 2007-02-27

**Authors:** P. Ravi Shankar

I read with interest the article by Mansfield et al. regarding educating health professionals about drug and device promotion [1]. Teaching about drug promotion is becoming increasingly important here in Nepal. During their pharmacology training at the Manipal College of Medical Sciences, Pokhara, medical students are taught to critically analyze drug advertisements and other promotional material against the World Health Organization's ethical criteria for medicinal drug promotion [2]. Their abilities in the critical analysis of drug promotional materials are evaluated during the pharmacology practical examination. The students are also taught to critically evaluate drug promotion by medical representatives (MRs) using the medium of role-play [3].

The four recommendations made by the authors are important, but developing countries may face problems in their implementation. We recommend that all health professionals be educated about decision making and evaluation of evidence and promotion. Our department runs a drug information center in the teaching hospital and we are trying to use this center to promote evidence-based medicine. However, there are no formal courses on evaluating the evidence. Doctors do not have an adequate knowledge of statistics to arrive at evidence-based decisions.

The authors' second and third recommendations pose further problems. Conferences in Nepal continue to be heavily sponsored by the pharmaceutical industry. MRs have unrestricted access to doctors in our hospital and in most other hospitals in Nepal. One-to-one visits, personal gifts, and other methods of sponsorship are the norm. Academic detailing is absent. I am personally ambivalent about banning one-to-one detailing. Many health professionals in South Asia are in private practice or work in small hospitals. It is an unfortunate fact, but MRs may be their only source of information about medicines. Banning MRs may deprive them of this source, however biased it may be. Exposing students to misleading presentation, fostering false beliefs, debunking these beliefs, and explaining the misleading techniques is an effective approach, used in our department during teaching critical evaluation of medicinal drug promotion.

We have had mixed success regarding educating health professionals to avoid promotion or look at it critically. We have been able to influence students during the first two years of their training. The influence of our training is considerably eroded once students are in their clinical phase. Enlisting the support of clinicians, making them aware of irrational promotion, and using their services to teach doctors in training is vital if we are to make progress. Education regarding the most reliable sources of information is lacking in South Asia. Health organizations, professional associations, and other bodies should develop information sources which are readily accessible to prescribers. Western information sources may have many limitations in developing countries.

So far, no medical student organizations in Nepal have taken up the issue of pharmaceutical promotion. The curriculum of Kathmandu University recommends teaching students to assess promotional materials. However, many medical schools do not address this vital issue. Meanwhile, the Nepalese pharmaceutical industry is coming of age. The pharmaceutical giants based in our Southern neighbor, India, are also active in Nepal. It is time for medical professionals to get their act together to ensure a proper relationship with the industry.
